# Supplementary material for: Tonoplast sucrose transporter SUT4‐dependent sugar partitioning modulates phenological transitions and reproductive success in poplar
Source: Plant J. 2025 Nov 16;124(4):e70554. doi: 10.1111/tpj.70554 (PMC12619991; doi:10.1111/tpj.70554)
Supplement: Supplementary file 3 — Table S1. Primers. Figure S1. Field trial timeline and temperature profiles. [file TPJ-124-0-s003.pdf]

Table S1. Primers

Name	Sequence (5’ to 3’)	Purpose
SUT5.U6	TCAAGCGAACCAGTAGGCTTGAGGTAGAAATGGTGGGTC	*SUT5*-KO construct assembly, with vector homology arms underlined
SUT5.SF	AACTTGCTATTTCTAGCTCTAAAACGACCCACCATTTCTACCTC	
SUT6.U6	TCAAGCGAACCAGTAGGCTTGAGGTAGAGATGGTGAGCA	*SUT6*-KO construct assembly, with vector homology arms underlined
SUT6.SF	AACTTGCTATTTCTAGCTCTAAAACTGCTCACCATCTCTACCTC	
SUT56.U6	TCAAGCGAACCAGTAGGCTTGTGAGGAGAGAGAGCTGCA	*SUT56*-KO construct assembly, with vector homology arms underlined
SUT56.SF	AACTTGCTATTTCTAGCTCTAAAACTGCAGCTCTCTCTCCTCA	
SUT56.tailF	CCTACACGACGCTCTTCCGATCTRCCGATTCGGGTACCGT	Amplicon sequencing, with Illumina adaptors underlined
SUT56.tailR	GTTCAGACGTGTGCTCTTCCGATCACCGCAGAGCCAAATGAA	

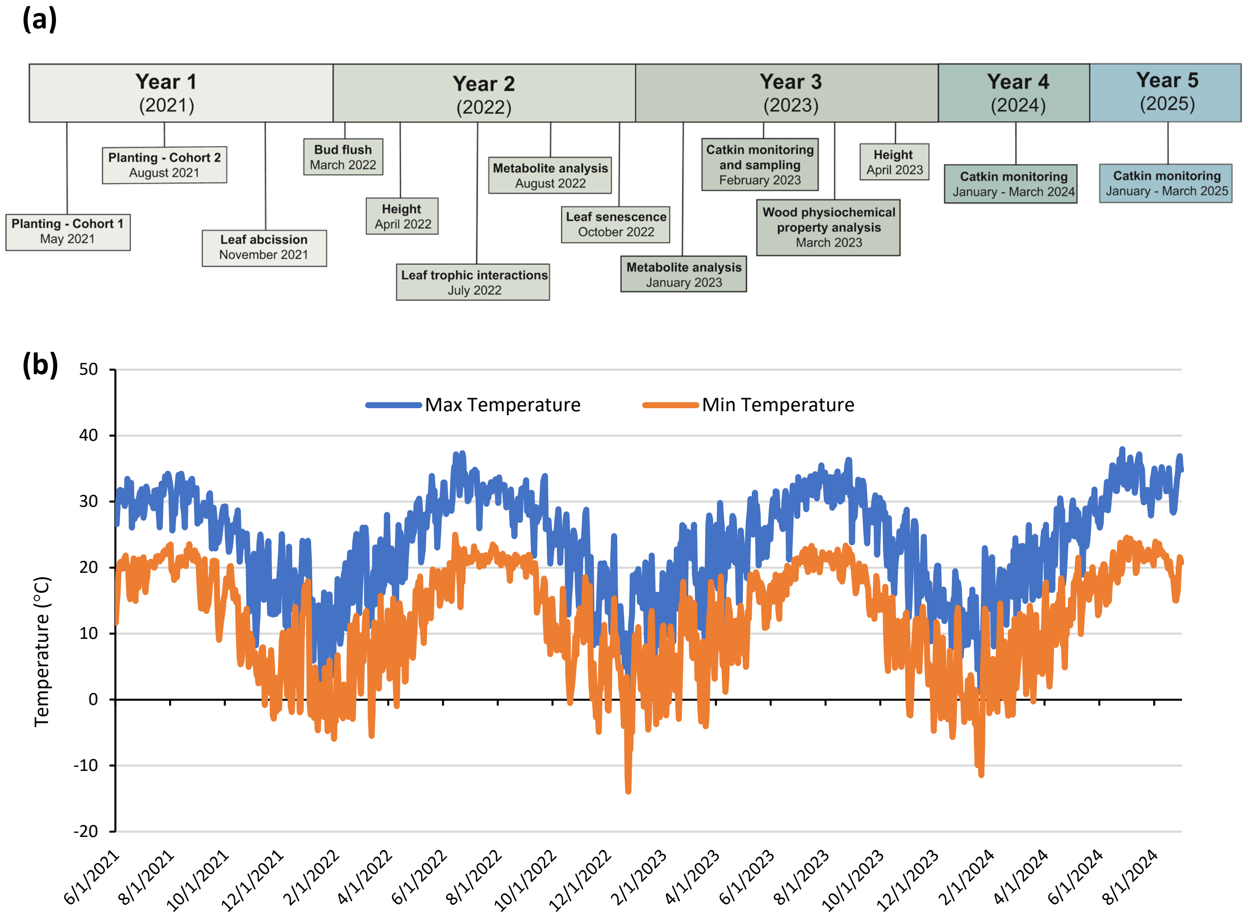

Figure S1. Field trial timeline and temperature profiles

(a) Timeline and schematic of various monitoring and sampling dates.

(b) Daily temperature profiles during the field trial obtained from http://www.georgiaweather.net.
